# Sampling methodology influences habitat suitability modeling for chiropteran species

**DOI:** 10.1002/ece3.10161

**Published:** 2023-06-09

**Authors:** Sarah M. Gaulke, Tara Hohoff, Brittany A. Rogness, Mark A. Davis

**Affiliations:** ^1^ Illinois Natural History Survey, Prairie Research Institute University of Illinois Urbana‐Champaign Champaign Illinois USA

**Keywords:** active detection, bats, eastern red bat, habitat suitability model, hoary bat, mist‐netting, passive detection, tri‐colored bat

## Abstract

Technological advances increase opportunities for novel wildlife survey methods. With increased detection methods, many organizations and agencies are creating habitat suitability models (HSMs) to identify critical habitats and prioritize conservation measures. However, multiple occurrence data types are used independently to create these HSMs with little understanding of how biases inherent to those data might impact HSM efficacy. We sought to understand how different data types can influence HSMs using three bat species (*Lasiurus borealis*, *Lasiurus cinereus*, and *Perimyotis subflavus*). We compared the overlap of models created from passive‐only (acoustics), active‐only (mist‐netting and wind turbine mortalities), and combined occurrences to identify the effect of multiple data types and detection bias. For each species, the active‐only models had the highest discriminatory ability to tell occurrence from background points and for two of the three species, active‐only models preformed best at maximizing the discrimination between presence and absence values. By comparing the niche overlaps of HSMs between data types, we found a high amount of variation with no species having over 45% overlap between the models. Passive models showed more suitable habitat in agricultural lands, while active models showed higher suitability in forested land, reflecting sampling bias. Overall, our results emphasize the need to carefully consider the influences of detection and survey biases on modeling, especially when combining multiple data types or using single data types to inform management interventions. Biases from sampling, behavior at the time of detection, false positive rates, and species life history intertwine to create striking differences among models. The final model output should consider biases of each detection type, particularly when the goal is to inform management decisions, as one data type may support very different management strategies than another.

## Graphical abstract

We compare habitat suitability models created from passive detection and active detection to show biases associated with the different data types. By comparing the niche overlaps of habitat suitability models between data types, we found a high amount of variation with no species having over 45% overlap between the models, emphasizing the need to consider the influences of detection and survey biases on modeling, especially when combining multiple data types.

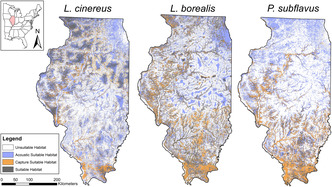

## 1 INTRODUCTION

Emerging technologies afford new opportunities for monitoring wildlife via passive or active detection. Passive detection, involving minimally invasive methods and often with no contact with the animal, includes tracking prints in snow or soil, listening to or recording calls, or camera trapping (Clare et al., 2017; Coxen et al., 2017; Sugai et al., 2019). More recently, environmental DNA (eDNA) and fecal sampling have yielded novel opportunities for passive detection (Beckmann et al., 2015; Harper et al., 2019; Hashemzadeh Segherloo et al., 2021). Active detection involves the direct capture or take and subsequent handling of individuals of species of interest (Coxen et al., 2017; Hohoff, 2016; Praca et al., 2009). These different detection methodologies occur across all types of wildlife; large carnivores can be passively detected via camera traps or actively captured (Bai et al., 2018; Kabir et al., 2017; Watts et al., 2019), birds can be surveyed passively through acoustic monitoring, or actively via mist‐netting (Coxen et al., 2017; Hallman, 2018; Neice & McRae, 2021), and fish are actively sampled through techniques electrofishing and can be passively sampled by watching through a fish ladder window (Mehdi et al., 2021; Rogers et al., 2003; Thompson et al., 2014).

Passive and active detection each have advantages and shortcomings. Particularly for cryptic species, passive detection can be more efficient and easier to deploy and may yield substantially more occurrences records at larger spatiotemporal scales (Clare et al., 2017; Coxen et al., 2017; Hohoff, 2016). While passive detection may have greater misidentification rates (e.g. misidentified acoustic calls, fuzzy camera trap photos, etc.), active detection can often be time‐consuming, expensive, and stressful to the organism with inherent mortality risk (Clare et al., 2017; Coleman et al., 2014; Russo & Voigt, 2016). Active sampling is frequently limited to one/few capture sites per trapping period, often with accessibility constraints, and dependent on species phenology (Rounsville et al., 2022; Zwart et al., 2014). In addition, for the extensive effort that some active capture methods require, few to no individuals may be captured (Bai et al., 2018; Flaquer et al., 2007). Different data collection methods have different detection biases that can impact downstream analyses (Clare et al., 2017; Flaquer et al., 2007; Risch et al., 2021). As each data type is biased, careful consideration of the purpose and sampling design to account for biases of each detection type is critical (Barnhart & Gillam, 2014; Ford et al., 2016; Risch et al., 2021).

Most occurrence data collected are used in modeling distributions, occupancy, habitat associations, or population trends. All occurrence data are inherently biased by the sampling paradigm, which can impact modeling, including HSM (Banner et al., 2018; Barnhart & Gillam, 2014; Gu & Swihart, 2004; Hallman, 2018). Passive data (e.g. acoustic, eDNA, etc.) are increasingly used in HSMs for multiple taxa, including fish, birds, and bats (Cox, 2019; Hallman, 2018; Hashemzadeh Segherloo et al., 2021). Higher misidentification rates associated with passive data can be minimized in some modeling frameworks, such as omitting imperfect detection and false positives; however, this can impact model precision and accuracy (Banner et al., 2018; Louvrier et al., 2019; Rojas et al., 2019). Combining both passive and active data sets may substantially increase the number of occurrences for cryptic species but introduce greater species misidentification and imperfect detection (Clare et al., 2017; Louvrier et al., 2019; Miller et al., 2011).

Landscape‐level research for Chiropteran species is necessary to identify critical habitats, patches, and corridors to target management interventions in the face of multiple compounding factors threatening North American bat populations (Bellamy et al., 2013; Rodhouse et al., 2019; Sandoval‐Herrera et al., 2020). While the impact of multiple detection types has been studied for occupancy modeling, there has been little research on landscape‐level effects of multiple data types on habitat modeling, particularly for chiropteran species (Banner et al., 2018; Clement et al., 2014; Rojas et al., 2019). Common detection methods for bats include passive detection (acoustics) or active (mist‐netting or wind turbine mortalities) (Barnhart & Gillam, 2014; Ford et al., 2016; Hohoff, 2016). Moving acoustic mobile transects are often called active detection for bat surveys; however, as this manuscript extrapolates beyond Chiropteran literature, all acoustics will be considered passive as there is no “in‐hand” assessment of the individual as there are for mist‐netting or wind‐turbine mortalities. Varying life histories and behaviors among Chiropteran species result in differential detection probabilities among sampling methods (Flaquer et al., 2007; Hohoff, 2016; O'Farrell & Gannon, 1999).

Using two different data types and mindful of their biases, we tested if data type (combined, passive, or active) impacts HSMs for three focal bat species (*Lasiurus borealis*, *Lasiurus cinereus*, *Perimyotis subflavus*). Our research questions were as follows: (1) Does sampling type yield different HSMs? (2) Does variation in species' ecology create differential HSMs derived from different sampling types? We hypothesize that the consistency across models from different data types will vary by species and their life‐history traits, that is, species with larger known disparities between passive and active detection will have greater model inconsistency. We predict the inconsistencies between models will reveal active models showing higher suitability in forested areas while passive models model suitable habitat in more agricultural areas.

## 2 METHODS

### 2.1 Study species

Midwestern bat species have diverse life‐history traits. The eastern red bat, *L. borealis* (Müller, 1776) is a common generalist forager that primarily roosts solitarily in foliage and tree bark (Limpert et al., 2007; Perry et al., 2007). Their long, narrow wings are adapted for fast flying over long distances and foraging in forest gaps, edges, and openings (Amelon et al., 2014; Starbuck et al., 2015; Walters et al., 2007). The hoary bat, *L. cinereus* (Palisot de Beauvois, 1796), roosts in trees, migrates long distances and primarily forages in open spaces, both behaviors linked with high wind‐turbine mortality (Friedenberg & Frick, 2021; Hayes et al., 2015; Weller et al., 2016). The tri‐colored bat, *P. subflavus* (Cuvier, 1832) has the longest hibernation time in Illinois, contributing to devastating losses from white‐nose syndrome (WNS) and prompting its candidacy for listing under the U.S. Endangered Species Act (Center for Biological Diversity & Defenders of Wildlife, 2016). They are foliage roosters during the summer and choose mature stands and forage above treetops and in partially open habitats (Farrow & Broders, 2011; O'Keefe, 2009; Veilleux et al., 2003).

### 2.2 Active detection data

We acquired historic capture data via a data sharing agreement with the Illinois Department of Natural Resources (IDNR) and the U.S. Fish and Wildlife Service (USFWS) for mist‐netting and summer wind farm mortalities from 1999 to 2021. Since IDNR primarily maintains records for threatened and endangered species, the historic capture data was incomplete for non‐listed species. Thus, we contacted IDNR's list of Illinois mist‐netting permittees requesting mist‐netting records for the three focal species between 1999 and 2021. We gathered eight responses, adding to the 46 mist‐net sites from 2015 to 2019 generated by the Illinois Bat Conservation Program (IBCP). We combined windfarm mortality and mist‐net records, reducing the number of individual records to one record per species per site. We removed sites with low positional, temporal accuracy, or no data associated with captures (*L. borealis*; *n* = 45, *L. cinereus*; *n* = 18, *P. subflavus*; *n* = 17).

### 2.3 Passive detection data

Acoustic data were collected by IBCP, following the North American Bat Monitoring Program (NABat) protocol for 20 NABat generalized random tessellation stratified (GRTS) cells surveyed annually from 2016 to 2020 (Illinois Bat Conservation Program, 2021; U.S. Geological Survey, 2021). A Song Meter SM4+ detector was deployed in 2–4 of the quadrants in each GRTS cell with a horizontally facing SMM‐U1 or U2 microphone 3 m above the ground (Wildlife Acoustics, n.d.). We chose sites to represent habitat diversity in each cell and deployed detectors for a minimum of four good weather nights (i.e., no rain, temperatures >15°C degrees, and sustained wind <13 kph) from 30 min before sunset to 30 min after sunrise, maintaining the same protocol for all years. IBCP also generated acoustic monitoring sites across Illinois that were surveyed with a similar acoustic protocol as the GRTS cells. These data are considered presence‐only; the survey effort at some sites (i.e., four nights) was insufficient to determine absence (Moreno & Halffter, 2000; Skalak et al., 2012).

We used the NABat protocol to process acoustic data in 2021 (Reichert et al., 2018). We processed all acoustic files through Sonobat 4 (Arcata, CA) using the medium filter to reduce noise files (Szewczak, 2010). Due to time and data storage constraints, we ran Kaleidoscope Pro 5.4.0 as the auto‐identifier (“Kaleidoscope Pro”, 2020). Kaleidoscope is a powerful auto‐classifier that identifies and provides maximum likelihood estimates of species occupancy. We considered α≤0.05 to be present and α≥0.05 to be absent following standard conservative protocols (Nocera et al., 2019; U.S. Fish and Wildlife Service & U.S. Geological Survey, 2019). These acoustic data were not manually vetted since NABat does not require manual vetting for data upload and observer bias has been identified with a qualitative review of bat acoustics as well (Fritsch & Bruckner, 2014). Manual review is not necessary to determine the absence of sensitive species according to USFWS regulations and this version of Kaleidoscope has over 86% positive ID rate for our three species of interest as well as an acceptable auto id software for MYSO and MYSE from a collaborative effort between USFWS and U.S. Geological Survey (U.S. Fish and Wildlife Service & U.S. Geological Survey, 2019).

### 2.4 Data cleaning

Active and passive data were combined and processed in R 4.1.2 (R Core Team, 2021). To reduce spatial autocorrelation, we removed records within 1 km of each other from the combined data set. Records were also temporally restricted from May 15 to August 15 to ensure that no records were from migration time periods following the USFWS Guidelines (U.S. Fish and Wildlife Service, 2020). After data cleaning and quality control, there were 88 acoustic and 176 capture occurrences for *L. borealis*, 86 acoustic and 30 capture occurrences for *L. cinereus*, and 28 acoustic and 78 capture occurrences for *P. subflavus* spread across Illinois.

### 2.5 Environmental layers

We used 16 land‐cover variables (Table S1) (per Cable et al., 2021) created from the Illinois Geospatial Clearing house land‐cover layer (Illinois Department of Natural Resources et al., 2003). Cable et al. (2021) used parallel methods to create a statewide HSM for the Indiana bat (*Myotis sodalis*). Cable used Fragstats (McGarigal & Marks, 1995) to find the number of patches of four land‐cover types, total area of eight land‐cover types, and total edge of two land‐cover types. Each metric was calculated at three different scales state‐wide (0.1, 0.5, and 1 km), representing roosting, foraging, and landscape distances.

An additional nine variables were also considered based on the top models of published chiropteran HSMs or occupancy models (Table S1). Many of these variables were forest stand structure metrics or topographical landscape metrics, gathered from publicly available GIS layers and resampled in ArcGIS for 100 m resolution to match layers (Esri Inc., 2021). Both temperature and precipitation layers were taken from a 30‐year normal and averaged across May–August to represent the summer average (PRISM Climate Group, 2021).

### 2.6 Modeling

For all variables, we created single‐predictor models in MaxEnt v3.4.4 using the combined (both active and passive) data types to choose the appropriate spatial scale for each variable and chose the top 15 variables for each species by AUC_test_ scores (Phillips et al., 2006, 2021). We chose to base the top variables off the combined data set as this minimized the habitat bias associated with each data type and created an opportunity for equal comparison between models of different data types. For univariate models, we used the default parameters with 20 replicates, and 10% random test percentage (Phillips et al., 2021). Using AUC_test_ scores, we determined the best spatial scale for each species for each land‐cover variable. We ranked the top scaled land‐cover variables among the non‐scaled variables, selecting the top 15 variables for each species with AUC_test_ > 0.5. A correlation matrix was created in ArcGIS for the 15 variables for each species and highly correlated variables (>0.7) were removed based on their univariate rank. With the remaining non‐correlated variables which were carried throughout the analysis, a global model was created for each species.

Using the global model, we tested differing regularization multipliers ranging from 1 to 12 using the same default parameters of 20 replicates and 10% as a random test percentage. The regularization multiplier with the top AUC_test_ scores was carried out through the rest of the analysis. Each species had three models ran with the same global model for each data set: passive‐only occurrences, active‐only occurrences, and combined passive and active detections. This yielded nine models across all species. All models were identically run in MaxEnt using presence‐only data, the top regularization multiplier, 20 replicates, 10% of datapoints withheld for testing from the data set, and 5000 maximum iterations. For pseudo‐absence points, MaxEnt randomly sampled 10,000 background points across the state using bootstrapping.

### 2.7 Model analysis

Model goodness‐of‐fit was assessed via AUC_test_ scores, maximum sum of the sensitivity plus specificity (maxSSS) scores, and omission rates. AUC_test_ scores predict the discriminatory ability of the model to tell occurrence points from background points with a score of 0.5 indicating that the model is no better than random chance and a score of 1 indicating perfect discriminatory ability (Jiménez‐Valverde, 2012). MaxSSS scores are values based on the maximum sum of the sensitivity (the proportion of correctly predicted presences) plus specificity (the proportion of correctly predicted background cells) logarithmic threshold to maximize discrimination between presence and absence values and have been shown to work well for presence‐only data (Liu et al., 2013). The data set from the opposite data types was used to test the omission rate which is the number of occurrences that are predicted outside suitable habitat. We used the following function in the Raster Calculator in ArcGIS to convert models from raw to log format, standardizing the suitability scale from 0 to 1 (Hammond et al., 2016).

$$\text{logistic}=\left(\mathrm{raw}*e^{\text{entropy}}\right)/\left(1+\mathrm{raw}*e^{\text{entropy}}\right)$$

To create binary models differentiating between suitable and unsuitable habitats, we created a threshold of omission for each species based on the bottom 10% of suitability scores for true presences in the test data sets (Hovick et al., 2015). For a comparison of the binary models, we ran a niche overlap function in ENMTools, using the Schoener's *D* value to calculate niche equivalence by the proportion of shared pixels between the two models (Schoener, 1968; Warren et al., 2010).

## 3 RESULTS

There was a range in the number of occurrences for each detection type among species (Table 1). *L. cinereus*, high‐flying open foragers, had almost three times as many passive detections (*n* = 86) than active detections (*n* = 30), whereas *P. subflavus*, a declining forest obligate, passive detections (*n* = 27) were much less frequent than active detections (*n* = 77). The *L. borealis*, a forest generalist, had the highest number of occurrences (*n* = 264) with a third of them being passive (*n* = 88) and two‐thirds being active (*n* = 176). For suitable habitat, forest‐related variables were dominant for *P. subflavus* and *L. borealis*, while open variables were dominant for *L. cinereus* (Table S2).

**TABLE 1:** Global model ran for each species and each data type.

Global model variables	Data type	Number of occurrences	AUC_Test_ value	% suitable habitat in Illinois	Omission rate (number of occurrences omitted)	MaxSSS
***Lasiurus borealis***						
Elevation + distance to water + existing vegetation height + quadratic mean diameter + area of agriculture in 0.1 km + area of bottomland forest in 1 km + area of forest in 0.1 km + area of water in 1 km + edge of forest in 0.1 km + edge of water in 1 km	Active	176	0.864	32.6%	54.6% (48)	0.3136
	Passive	88	0.812	43.3%	53.4% (94)	0.4701
	Combined	264	0.791	50.9%	–	0.4641
***Lasiurus cinereus***						
Aspect + distance to roads + distance to water + existing vegetation height + number of patches of forest in 0.1 km + stand density index + solar radiation + area of agriculture in 0.1 km + area of bottomland forest in 0.1 km + area of open canopy deciduous forest in 1 km + area of urban in 0.1 km + area of water in 1 km	Active	30	0.839	25.4%	72.1% (62)	0.4794
	Passive	86	0.817	47%	46.7% (14)	0.4642
	Combined	116	0.803	46.9%	–	0.4198
***Perimyotis subflavus***						
Elevation + distance to roads + existing vegetation height + number of patches of forest in 100 m + number of patches of water in 500 m + quadratic mean diameter + stand density index + area of agriculture in 500 m + area of bottomland forest in 1 km + area of water in 500 m	Active	77	0.921	19.8%	78.6% (22)	0.2112
	Passive	27	0.755	33%	61.5% (48)	0.4846
	Combined	104	0.852	28.3%	–	0.303

*Note*: AUC_test_ values show each model's goodness‐of‐fit. The percent of suitable habitat is the percent of Illinois that has been found as suitable after the binary threshold. The omission rate is the percent of opposite data type points modeled in unsuitable habitat in the binary model with the number of occurrences omitted in parentheses, that is, the active data type's omission rate is the percent of passive occurrences that were omitted.

**FIGURE 1:**
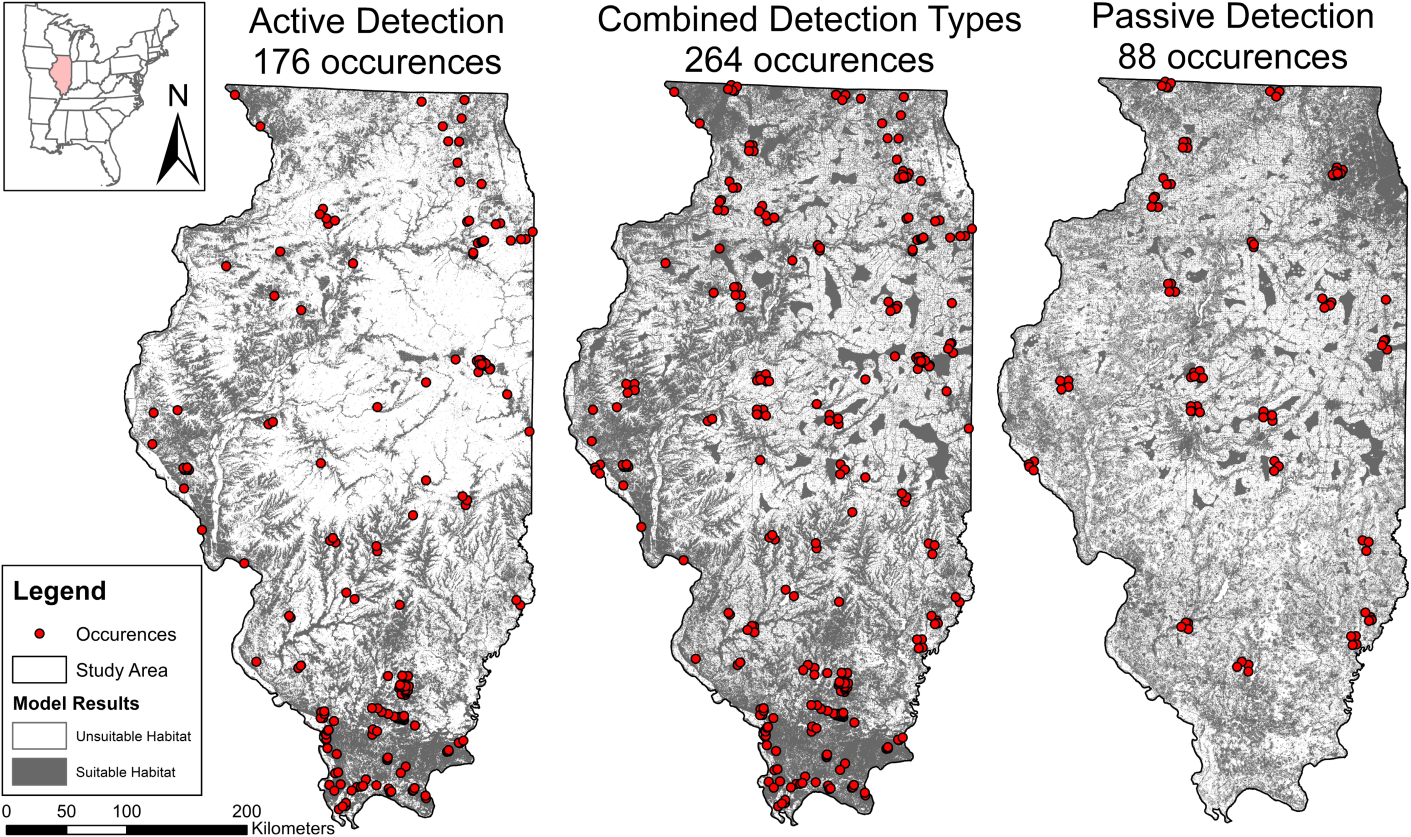
Habitat suitability models for *Lasiurus borealis* with detection types; active, combined, and passive in Illinois, United States.

Model outputs differed greatly between data types; however, models based on active detection had the highest AUCs for all species and two of the three highest maxSSS scores (Table 1). For *L. borealis*, models based on active detection had the highest AUC (0.86), lowest maxSSS score (0.21), highest number of occurrences, and projected the most conservative HSM (Table 1). Only 32.6% of the state was considered suitable habitat compared with 43.3% with passive detection and 50.9% with a combined data set. The omission rate between the two data types (53–54%) was high for *L. borealis*. The active HSM models revealed suitable habitat in southern Illinois and following forested riparian areas and major river corridors, while the passive HSM revealed most suitable habitat near the Chicago region (the northeast corner of Illinois) and in large contiguous agricultural patches in the central western part of the state (Figure 1 and Figure S1). The combined model merged these agricultural patches with forested/riparian suitability but predicted Chicago as unsuitable habitat.

For *L. cinereus*, the active model had the highest AUC value (0.84) and maxSSS score (0.48) and the most conservative HSM with only 25% of the state identified as suitable habitat (Table 1). While the combined and passive models had AUC values very close to the active model (0.80 and 0.82, respectively), the amount of modeled suitable habitat nearly doubled, with 47% of the state comprised of suitable habitat in both models. While the number of active detections was one‐third of the overall number of detections, the omission rate of the active model was high at 72% of passive occurrences being modeled in unsuitable habitat. The passive model had a lower omission rate of 46.7% which is high.

**FIGURE 2:**
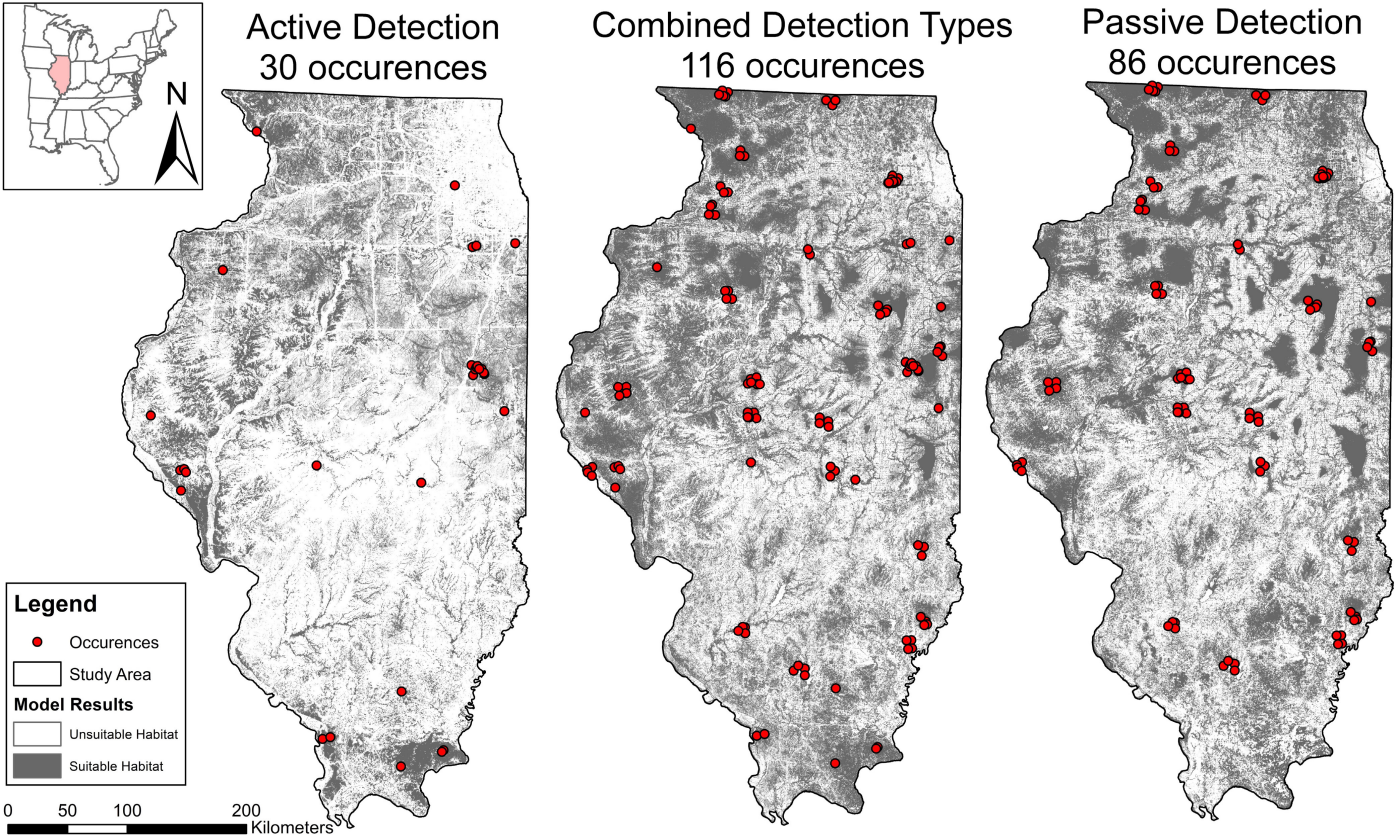
Habitat suitability models for *Lasiurus cinereus* with detection types; active, combined, and passive in Illinois, United States.

Active HSMs for *L. cinereus* revealed small suitable patches distributed across northwestern Illinois with a large habitat patch in the southern forests (Figure 2). In addition, major roads, highways, and waterways stand out in this model as unsuitable habitat. The passive HSM modeled much larger‐scale, evenly distributed and contiguous patches, particularly in agricultural areas. The southern third of the state included more fine‐scale suitable habitat, but the influence of roads and waterways were less pronounced than in the active model. The model with combined detections included both the larger‐scale patches and greater distribution of the model based on passive detections with the smaller‐scale suitability of the active model.

For *P. subflavus*, the active model had a high AUC value of 0.9212 and 19.8% of the state modeled as suitable habitat (Table 1). The passive model yielded the lowest AUC value of 0.75 and highest maxSSS score of 0.47 with 33% of the state as suitable habitat while the combined model had an AUC value of 0.85 with 28.3% of the state as suitable habitat. Both omission rates of *P. subflavus* were high with over 61% of occurrences omitted in the passive model and over 78% of passive occurrences omitted in the active model.

**FIGURE 3:**
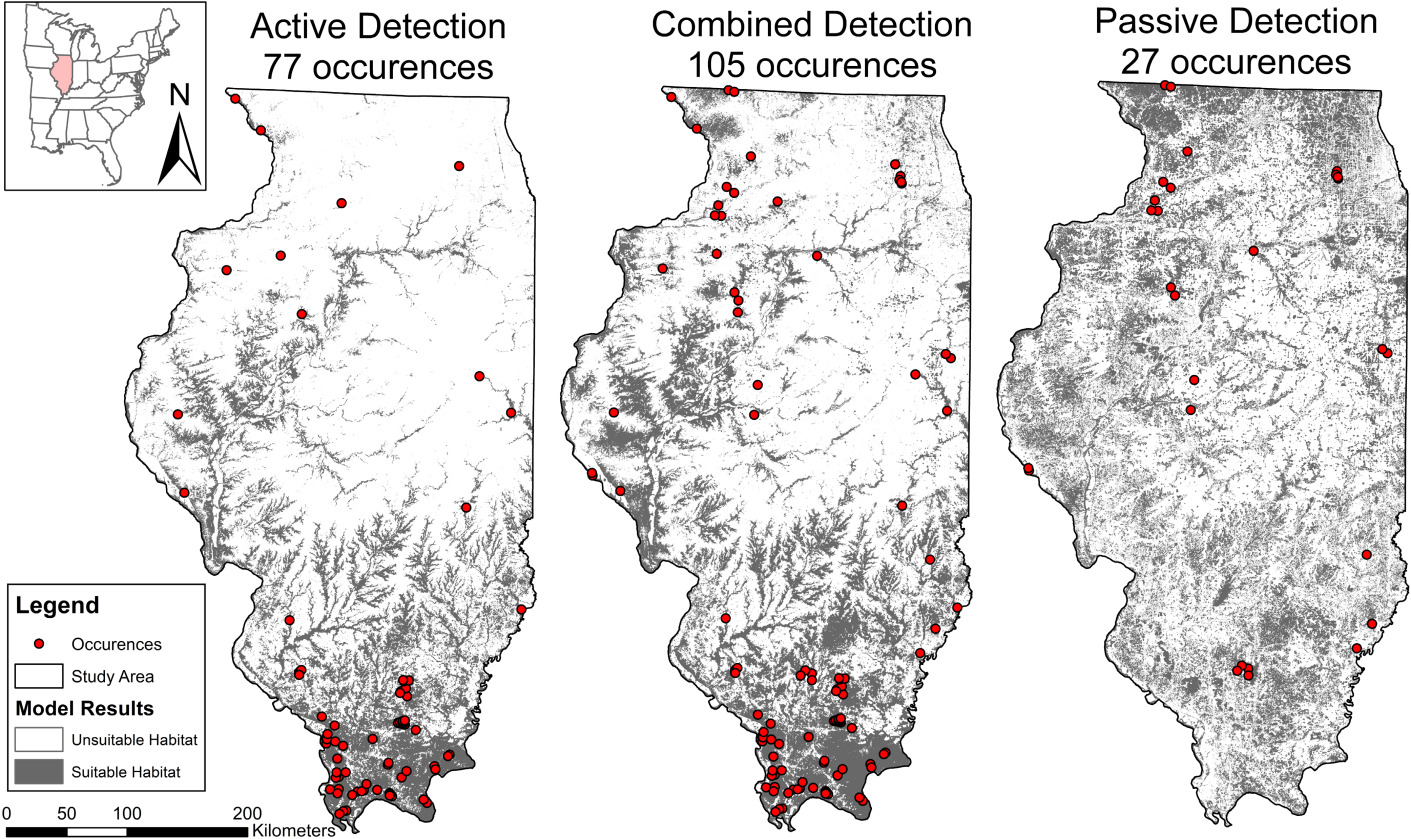
Habitat suitability models for *Perimyotis subflavus* with detection types; active, combined, and passive in Illinois, United States.

*Perimyotis subflavus* HSMs are dissimilar in predicted suitable habitat (Figure 3). The active HSM confined the suitable habitat to forested and major riparian zones throughout the state, particularly in and around Shawnee National Forest. Conversely, the passive model revealed no obvious forest association and an even distribution throughout the state, with a slight increase in suitable habitat in the northern third. The wide distribution from the passive model was not reflected in the combined model in areas of dense agriculture.

**FIGURE 4:**
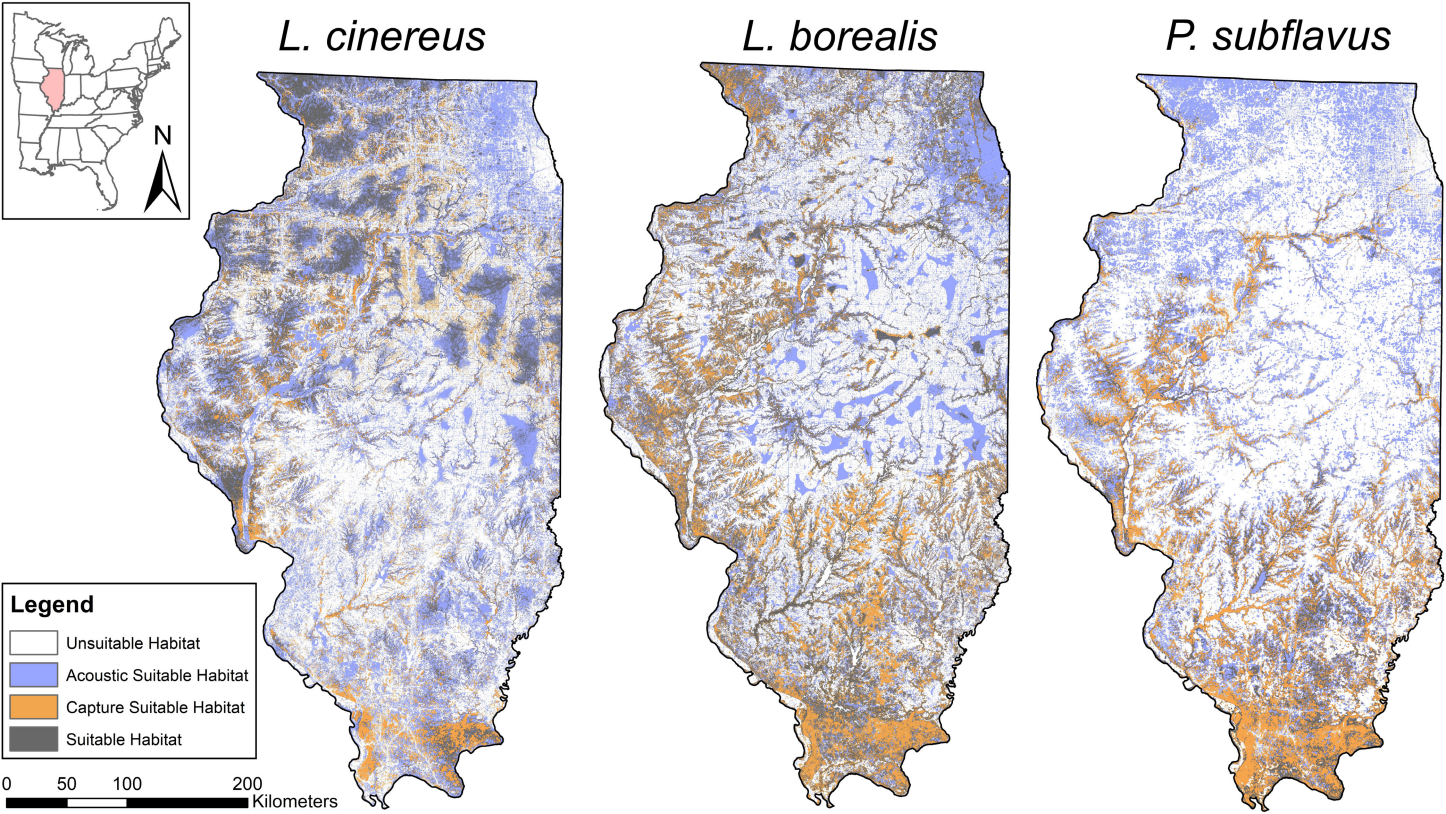
Maps showing the difference between passive and active models for *Lasiurus cinereus*, *Lasiurus borealis*, and *Perimyotis subflavus in Illinois, United States*. Orange indicates suitable areas only predicted by the active model, while purple indicates areas that only the passive model found suitable. Gray areas are suitable habitat predicted by both the active and passive model.

Comparing niche overlap values for different model types showed a maximum of 45% (range: 22–45%) overlap between active and passive detection types across all species (Table 2), indicative of substantial disparity between the two data types among species. In fact, >50% of the entire state of Illinois was modeled differently when active and passive models were compared (Figure 4). Comparing models from passive detections to models from combined detections resulted in a higher degree of overlap (range: 48–83%) and active models to the combined models (range: 43–63%). For all species, the data type with larger sample size had greater overlap with the combined model. This is expected as the larger sample size would have a higher influence on the distribution.

**TABLE 2:** Niche overlap matrix for the three focal species with Schoener's D calculating niche similarity from 0 to 1 between each model.

Species	Data types		
	Active versus passive	Active versus combined	Passive versus combined
*Lasiurus borealis*	0.4507	0.6245	0.7141
*Lasiurus cinereus*	0.3400	0.4385	0.8355
*Perimyotis subflavus*	0.2233	0.6327	0.4898

## 4 DISCUSSION

As technological advances have introduced efficiencies and opportunities for measuring and monitoring biodiversity, understanding and comparing the trade‐offs and biases between conventional, active sampling approaches is critical to ensure the quality of conclusions made from these analyses (Barnhart, 2014; Clare et al., 2017; Ford et al., 2016). As eDNA, acoustic monitoring, camera trapping, and other passive methods are deployed, researchers must be confident in the data to inform management decisions. Here we compared passive and active sampling for bats. Across all species, the maximum overlap of 45% difference in HSMs between passive and active indicates that sampling bias between data types influences HSMs, with important implications for informing management decisions (Table 2). The active model had the highest maxSSS for two of the three species, highest AUC_Test_ value, and the smallest amount of suitable habitat for all species (Table 1). This indicates that active data may create more robust HSM for chiropteran species on a landscape scale, even for species that are more difficult to capture, contradicting previous studies (Barnhart & Gillam, 2014; Ford et al., 2016). However, using an active model for conservation decisions may overemphasize forested habitat and underemphasize open foraging and flying habitat. Overall, this underscores that considerations should be made regarding the influence of data types on HSMs in three key ways: sampling bias, biological bias during detection, and the species bias from their life history and ecology.

### 4.1 Sampling bias

Sampling bias for each data type is visible in the models, as the efficacy of different detection types varies between locations (Gu & Swihart, 2004; Zwart et al., 2014). For effective mist‐netting of chiropteran and avian species, nets need to be placed in flyways and corridors that funnel animals into the net (Geluso & Geluso, 2012; MacCarthy et al., 2006; O'Farrell & Gannon, 1999). Forests and riparian zones provide these “funnels” and are frequent netting sites (Coleman et al., 2014; Geluso & Geluso, 2012). As such, mist‐net derived occurrences are distributed primarily in riparian and forested zones and lacking in residential and open fields (e.g. prairie, row crop agriculture, etc.). This creates sampling bias with higher numbers of active occurrences within forested and riparian zones, which is reflected in our active models and illustrated by the majority of suitable habitat falling along Illinois' big rivers. This may be the reason for active data having the lowest percentage of the state as suitable for all focal species, as only 12% of Illinois is forested and may diminish with increasing cell size and land‐cover type homogenizes (Luman et al., 2004).

Conversely, passive data are easily collected, especially in urban environments, open fields, row agriculture, forests, and riparian habitats. While vegetation reduces the distance to detection for acoustics and camera trapping, other passive methods such as eDNA and fecal sampling are less impacted by land‐cover type (Leempoel et al., 2020; Moll et al., 2020; O'Keefe et al., 2014). Passive sampling was distributed more broadly in our study and evenly throughout the landscape, although with a critical disparity in Illinois' southern forests. The lack of acoustics in Illinois' forested regions mainly occurred because there were no NABat GRTS cells allocated in this portion of the state as there is minimal forest in Illinois, so by random chance none were selected (Loeb et al., 2015; U.S. Geological Survey, 2022). This manifests in greater distribution of suitable habitat as more of the state land cover is represented, not strictly confined to forested areas of the state as in the active models. To a degree, this may reflect, the open habitat proclivities of more generalist species (e.g., *L. borealis* and *L. cinereus*), which is supported by previous habitat modeling and minimize the habitat proclivities of clutter adapted species (e.g., *P. subflavus*) (Menzel et al., 2005; Vanausdall et al., 2018; Wieringa et al., 2021). For a rarer and shorter distance forest specialist such as *P. subflavus*, the passive model does not reflect their forest requirements, potentially because of a lack of sampling in the predominantly forested region of the state compared with the longer range and more generalist *L. borealis* and *L. cinereus*. (Cox, 2019; Farrow & Broders, 2011; Schaefer, 2017).

### 4.2 Biological bias on modeling

Previous studies leveraging multiple data types for chiropteran HSMs found a significant difference among data types and species. Ford et al. (2016) modeled an HSM for the northern long‐eared bat (*Myotis septentrionalis*) (Trouessart, 1897) at a local scale and strongly recommended against combining three data types, (i.e., acoustics, mist‐netting, and roost locations), as it masked ecological trends of roosting versus foraging (Ford et al., 2016). While this model was at a local scale and used an additional data type (roost sites), their concerns about defining the model objective and using data to reflect that model objective remain valid for all model analyses. Roost locations constrict occurrence locations to suitable roosting habitat and may sway the model from a generalist model to a more ecologically specific one, ostensibly limiting the inferential efficacy of a general, landscape‐level model.

Our model created a general summer use HSM from passive and active sampling, as both types of detection used are evidence of general bat use on the landscape. Some data types, such as roosting data for bats, are record locations for a specific ecological need. For other data types like passive and active detection for bats and other species, it can often be unclear what the animal is doing when detected (e.g., migrating, looking for a mate, foraging, or commuting) (Ford et al., 2016). Even using a single data type leaves uncertainty as to what the species are doing when detected, and thus these models reflect broad habitat associations. Without a clear association of the animal's biological context at each occurrence, incorporating multiple detection types can generalize the model's ecological utility, which may or may not be beneficial based on the modeling goal(s). This may not be the case for other species, where occurrence type may reflect a specific biological need (e.g. roosting, nesting, spawning locations, or a species that vocalizes only during mating season). Using that data type would result in a specific, biologically meaningful HSM in these contexts.

### 4.3 Species bias

Barnhart and Gillam (2014) modeled landscape‐scale HSMs for six bat species in North Dakota, pairing passive and active sampling. They found that the best data type was species‐specific, suggesting researchers should carefully consider each species' life history and ecology to select appropriate sampling methods for optimizing HSMs, even if that meant combining data sets (Barnhart & Gillam, 2014). We used a larger occurrence data set with over 100 (vs. 17 in Barnhart & Gillam, 2014) sites for each species, better representing a landscape‐scale sampling protocol and revealing different trends than Barnhart and Gillam (2014). The difference between our findings and their results may be attributed to different genera, as none of our focal species were *Myotis*, which were the only species in which passive did better than active in Barnhart and Gillam (2014). We echo their findings that species life history and ecology should be strongly considered when building a sampling design and creating HSMs, as the impact of detection differences varied between our three focal species.

One example of the impact of species ecology is the potential difference in the number of occurrences between common species that are easily detected and cryptic species, as the increasing sample size is linked to improved model performance (Hirzel & Guisan, 2002). For cryptic, difficult‐to‐detect species, we can increase HSM robustness by using multiple methods and increasing sample size (Clare et al., 2017; Coxen et al., 2017; Hohoff, 2016). Alternatively, timing sampling to maximize detections of specific life stages may improve model efficacy (Kunz & Hood, 2000; Moss et al., 2022; Smith et al., 2006). For example, in amphibians, the efficacy of eDNA to accurately estimate occupancy decreased as amphibians metamorphosed, underperforming conventional, active sampling (Moss et al., 2022). Species life history plays a vital role in detection and number of occurrences and as such the best data type may fluctuate among species, across life stages and time periods. *P. subflavus* populations have dramatically declined from WNS since 2006 (Cheng et al., 2021). Our study uses active records from pre‐ and post‐WNS while our passive records are primarily from post‐WNS. While the active model represents habitat that the species may restore with more robust population numbers, the passive model may represent a limited range of the current smaller population size and this may contribute to the lower score of the passive model. Overall, the low number of occurrence records is a limitation of our study as some of the data sets (i.e., passive data for *P. subflavus*) had relatively few occurrences given the area of modeling. While Maxent has been shown to perform well for small data sets (Pearson et al., 2007), maintaining 10% of records for testing our univariate models was a limited number of occurrences for some species and may have impacted our low parameter scores.

A significant drawback of passive sampling that was unaccounted for in this study was false positives, which may be particularly important for *L. borealis* and *P. subflavus*. Using maximum likelihood estimate values for each site as we did helps to minimize false positives in the data set; however, there remains a potential impact. In acoustics, false‐positive and false‐negative rates vary by species and are lower for species with distinctive call shapes and frequencies, and higher for species with similar calls (Clement et al., 2014; Rojas et al., 2019; Russo & Voigt, 2016). While manual vetting may reduce false‐positive rates, subjectivity among various call ID software and specialists remains (Fritsch & Bruckner, 2014; Russo & Voigt, 2016). This extends beyond acoustics to include misidentification of feces, incorrect track identification, and blurry camera trap photos, where genetic analyses provide confidence for some detection methods (Clare et al., 2017; Guan et al., 2020; Louvrier et al., 2019). For species with high false identification rates, occupancy models have begun incorporating false‐positive parameterizations, thus improving model estimation (Clement et al., 2014; Miller et al., 2011; Rojas et al., 2019). Incorrect species identification may contribute to poor model performance for passive sampling data. Therefore, accounting for false positives and negatives is a critical consideration in modeling exercises leveraging these data types.

### 4.4 Management considerations

From a management perspective, the differences among models are striking and call for careful consideration when leveraging their outputs to inform decision‐making and management interventions. Such HSMs are often used in management interventions and conservation planning, including setting restoration objectives, purchasing land for protection, identifying potential areas to improve connectivity, or siting future wind energy developments (Cable et al., 2021; Stevens & Conway, 2020; Vanausdall et al., 2018). Additionally, HSMs are frequently used to predict the potential impacts of climate change on species' distributional boundaries (Coxen et al., 2017; Davis et al., 2015; Razgour et al., 2016). Comparing HSMs based on different data types or combined data sets that are heavily skewed towards one data type may highlight differences due to sampling bias and detection probabilities rather than actual ecological/environmental impacts. Thus, different management decisions might be made based on passive versus active HSMs. Caution is warranted when comparing HSMs, and model biases must be considered when drawing inferences, especially when used in a management context. While we chose the variable spatial scale and top 15 variables based on univariate models from the combined data set, different variable choices may have helped to alleviate some of the model bias between data types as different variables and variable scales could have converged on models with higher similarity. We maintain that using the combined data set creates the best opportunity for model comparison; however, for management purposes or conservation decisions, different variables may have been selected creating different models. While this study focuses on bats, passive and active detections are common differences in surveying many species leading to the broad applicability of this study. The three factors discussed; sampling bias, behavior at time of detection, and species life history, are inextricably linked. As such, consideration of each will create more biologically relevant and robust models, while simultaneously promoting improved management outcomes.

## AUTHOR CONTRIBUTIONS

**Sarah Gaulke:** Conceptualization (lead); data curation (lead); formal analysis (lead); investigation (lead); methodology (lead); project administration (lead); resources (equal); software (lead); supervision (lead); validation (lead); visualization (lead); writing – original draft (lead); writing – review and editing (equal). **Tara Hohoff:** Data curation (equal); funding acquisition (lead); methodology (supporting); resources (supporting); supervision (supporting); writing – review and editing (supporting). **Brittany A. Rogness:** Data curation (equal); investigation (equal); resources (equal); writing – review and editing (supporting). **Mark A. Davis:** Conceptualization (supporting); formal analysis (supporting); funding acquisition (lead); methodology (supporting); project administration (supporting); resources (supporting); supervision (supporting); writing – review and editing (equal).

## CONFLICT OF INTEREST STATEMENT

The authors have no conflict of interest to disclose.

## Supporting information


Appendix S1

## Data Availability

The binary HSMs are available on Dryad at 10.5061/dryad.t1g1jwt6r. The occurrence records are not available as they are sensitive locations.
