# Supplementary material for: Sampling methodology influences habitat suitability modeling for chiropteran species
Source: Ecol Evol. 2023 Jun 9;13(6):e10161. doi: 10.1002/ece3.10161 (PMC10256621; doi:10.1002/ece3.10161)
Supplement: Supplementary file 1 — Appendix S1 [file ECE3-13-e10161-s001.docx]

**Supplementary Materials**

Table S1. Variables tested in univariate models for the eastern red, hoary, and tri-colored bat.

| **Variables** | **Spatial scale (km)** | **Original data source** | **Creator** |
| --- | --- | --- | --- |
| Aspect | 0.1 | (U.S. Geological Survey 2018) | Gaulke |
| Canopy Cover | 0.1 | (U.S. Geological Survey 2018) | Gaulke |
| Elevation | 0.1 | (U.S. Geological Survey 2018) | Gaulke |
| Distance to Roads | 0.1 | (Illinois Department of Transportation 2018) | (Cable *et al.* 2021) |
| Distance to Water | 0.1 | (U.S. Geological Survey 2020) | (Cable *et al.* 2021) |
| Existing Vegetation Height | 0.1 | (U.S. Department of Interior *et al.* 2013) | Gaulke |
| Number of Patches of Agriculture | 0.1, 0.5, 1 | (Illinois Department of Natural Resources *et al.* 2003) | (Cable *et al.* 2021) |
| Number of Patches of Forest | 0.1, 0.5, 1 | (Illinois Department of Natural Resources *et al.* 2003) | (Cable *et al.* 2021) |
| Number of Patches of Urban | 0.1, 0.5, 1 | (Illinois Department of Natural Resources *et al.* 2003) | (Cable *et al.* 2021) |
| Number of Patches of Water | 0.1, 0.5, 1 | (Illinois Department of Natural Resources *et al.* 2003) | (Cable *et al.* 2021) |
| Null | 0.1 | (Illinois Department of Natural Resources *et al.* 2003) | (Cable *et al.* 2021) |
| Precipitation (30-year average of monthly summer precipitation) | 0.1 | (PRISM Climate Group 2021) | Gaulke |
| Quadratic Mean Diameter | 0.1 | (U.S. Forest Service 2012) | Gaulke |
| Stand Density Index | 0.1 | (U.S. Forest Service 2012) | Gaulke |
| Solar Radiation | 0.1 | (Solargis 2019) | Gaulke |
| Total Area of Agriculture | 0.1, 0.5, 1 | (Illinois Department of Natural Resources *et al.* 2003) | (Cable *et al.* 2021) |
| Total Area of Bottomland Forest | 0.1, 0.5, 1 | (Illinois Department of Natural Resources *et al.* 2003) | (Cable *et al.* 2021) |
| Total Area of Closed Canopy Deciduous Forest | 0.1, 0.5, 1 | (Illinois Department of Natural Resources *et al.* 2003) | (Cable *et al.* 2021) |
| Total Area of Coniferous Forest | 0.1, 0.5, 1 | (Illinois Department of Natural Resources *et al.* 2003) | (Cable *et al.* 2021) |
| Total Area of Forest | 0.1, 0.5, 1 | (Illinois Department of Natural Resources *et al.* 2003) | (Cable *et al.* 2021) |
| Total Area of Open Canopy Deciduous Forest | 0.1, 0.5, 1 | (Illinois Department of Natural Resources *et al.* 2003) | (Cable *et al.* 2021) |
| Total Area of Urban | 0.1, 0.5, 1 | (Illinois Department of Natural Resources *et al.* 2003) | (Cable *et al.* 2021) |
| Total Area of Water | 0.1, 0.5, 1 | (Illinois Department of Natural Resources *et al.* 2003) | (Cable *et al.* 2021) |
| Total Edge of Forest | 0.1, 0.5, 1 | (Illinois Department of Natural Resources *et al.* 2003) | (Cable *et al.* 2021) |
| Total Edge of Water | 0.1, 0.5, 1 | (Illinois Department of Natural Resources *et al.* 2003) | (Cable *et al.* 2021) |
| Temperature (30-year average of monthly minimum summer temperature) | 0.1 | (PRISM Climate Group 2021) | Gaulke |

Table S2. Univariate model results ranked by discriminant ability of the Area Under the Curve Test (AUC_test_) values based on combined occurrence points (i.e. both acoustic and capture) for the eastern red, hoary, and tri-colored bat across Illinois collected from 1999-2021. The top 15 variables were used in subsequent multivariate models.

| **Rank** | **Eastern red** | **AUC** | **Hoary** | **AUC** | **Tri-colored** | **AUC** |
| --- | --- | --- | --- | --- | --- | --- |
| 1 | Total area of ag in 0.1km | 0.7231 | Total area of ag in 0.1km | 0.6376 | Distance to roads | 0.9056 |
| 2 | Total area of forest in 0.1km | 0.7206 | Distance to roads | 0.6172 | Stand density index | 0.8071 |
| 3 | Existing vegetation height | 0.7193 | Total area of urban in 0.1km | 0.6038 | Total area of bottomland forest in 1km | 0.8033 |
| 4 | Stand density index | 0.7169 | Distance to water | 0.5968 | Total area of forest in 0.5km | 0.798 |
| 5 | Number of patches of forest in 0.1km | 0.7016 | Number of patches of forest in 0.1km | 0.5871 | Existing vegetation height | 0.7959 |
| 6 | Quadratic mean diameter | 0.6999 | Total area of water in 1km | 0.586 | Total area of ag in 0.5km | 0.7858 |
| 7 | Canopy cover | 0.6985 | Solar radiation | 0.5819 | Elevation | 0.7721 |
| 8 | Total edge of forest in 0.1km | 0.6967 | Existing vegetation height | 0.5812 | Quadratic mean diameter | 0.7719 |
| 9 | Total area of bottomland forest in 1km | 0.6548 | Total area of forest in 0.1km | 0.579 | Canopy cover | 0.7678 |
| 10 | Total edge of water in 1km | 0.647 | Total edge of forest in 0.1km | 0.5741 | Total area of water in 0.5km | 0.762 |
| 11 | Elevation | 0.6434 | Stand density index | 0.573 | Temperature | 0.7502 |
| 12 | Total area of water in 1km | 0.6412 | Aspect | 0.57 | Total edge of forest in 0.5km | 0.7444 |
| 13 | Number of patches of water in 1km | 0.6355 | Number of patches of urban in 0.1km | 0.5611 | Number of patches of forest in 0.1km | 0.7362 |
| 14 | Temperature | 0.6258 | Total area of open canopy deciduous forest in 1km | 0.5568 | Number of patches of water in 0.5km | 0.7313 |
| 15 | Distance to water | 0.6243 | Total area of bottomland forest in 0.1km | 0.5554 | Total edge of water in 0.5km | 0.7134 |
| 16 | Number of patches of agriculture in 0.5km | 0.6186 | Total edge of water in 1km | 0.5509 | Total area of closed canopy deciduous forest in 1km | 0.7124 |
| 17 | Total area of closed canopy deciduous forest in 1km | 0.6126 | Temperature | 0.5501 | Total area of open canopy deciduous forest in 1km | 0.6996 |
| 18 | Distance to roads | 0.611 | Total area of closed canopy deciduous forest in 0.1km | 0.5447 | Number of patches of agriculture in 1km | 0.6993 |
| 19 | Solar radiation | 0.6039 | Precipitation | 0.5421 | Solar radiation | 0.6782 |
| 20 | Total area of open canopy deciduous forest in 1km | 0.5914 | Canopy cover | 0.5418 | Precipitation | 0.6563 |
| 21 | Precipitation | 0.5723 | Number of patches of agriculture in 1km | 0.5271 | Distance to water | 0.637 |
| 22 | Aspect | 0.5719 | Quadratic mean diameter | 0.5261 | Total area of coniferous forest in 0.5km | 0.6255 |
| 23 | Total area of coniferous forest in 1km | 0.5475 | Number of patches of water in 0.1km | 0.5259 | Aspect | 0.5804 |
| 24 | Null | 0.5388 | Elevation | 0.5244 | Number of patches of urban in 1km | 0.5566 |
| 25 | Total area of urban in 0.1km | 0.5278 | Total area of coniferous forest in 1km | 0.5136 | Total area of urban in 0.1km | 0.5504 |
| 26 | Number of patches of urban in 0.5km | 0.52 | Null | 0.4507 | Null | 0.5279 |


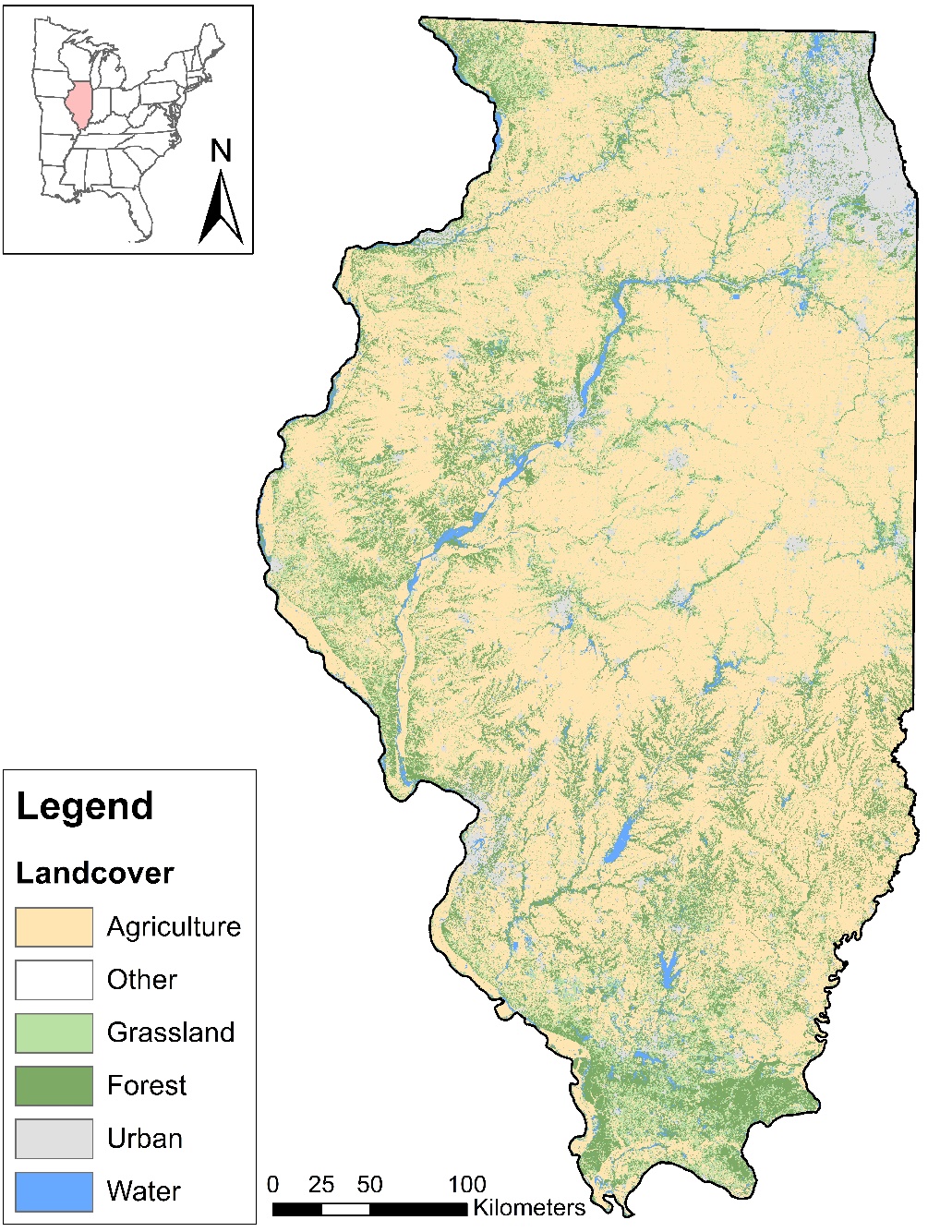


Figure S1. Landcover of Illinois, United States.

**Works Cited**

Illinois Department of Natural Resources, Illinois Natural History Survey, Illinois State Geological Survey, Illinois Department of Agriculture & United States Department of Agriculture National Agricultural Statistics Service. (2003). Land Cover of Illinois 1999–2000 Data. <https://clearinghouse.isgs.illinois.edu/data/land-cover/land-cover-illinois-1999-2000-data>

Illinois Department of Transportation. (2018). Illinois Technology Transfer Center. <https://apps.dot.illinois.gov/gist2/>

PRISM Climate Group. (2021). PRISM Climate Data.

Solargis. (2019). Solargis global solar model. <https://solargis.com/maps-and-gis-data/download/usa>.

U.S. Department of Interior, U.S. Geological Survey & U.S. Department of Agriculture. (2013). LANDFIRE: Existing Vegetation Height. <https://www.landfire.gov/>

U.S. Forest Service. (2012). Individual tree species parameter maps. <https://www.fs.fed.us/foresthealth/applied-sciences/mapping-reporting/indiv-tree-parameter-maps.shtml>

U.S. Geological Survey. (2018). GAP analysis project: Species and ancillary data. In *Gap analysis project species range maps CONUS_2001*. U.S. Geological Survey Data Release.

U.S. Geological Survey. (2020). National Hydrography Dataset (NHD). <https://nhd.usgs.gov/>
